# Complete Mitochondrial Genome of Two *Amathusiini* Species (Lepidoideae: *Nymphalidae*: *Satyrinae*): Characterization, Comparative Analyses, and Phylogenetic Implications

**DOI:** 10.3390/genes16040447

**Published:** 2025-04-12

**Authors:** Qinghui Shi, Xinyue Wang, Jianhong Xing, Xiaoyun Xu, Gang Sun, Juncheng Zhang

**Affiliations:** 1 Fujian Provincial Key Laboratory of Resources and Environment Monitoring & Sustainable Management and Utilization, Sanming University, Sanming 365004, China; ahui0214@sina.com (Q.S.); 19193807039@163.com (X.W.); jianhong_xing@126.com (J.X.); 18250582105@163.com (X.X.); sungang@nenu.edu.cn (G.S.); 2 Medical Plant Exploitation and Utilization Engineering Research Center, Sanming University, Sanming 365004, China

**Keywords:** *Satyrinae*, *Discophora sondaica*, *Aemona amathusia*, mitochondrial genome, phylogeny, comparative analysis

## Abstract

**Background**: The *Satyrinae* subfamily represents a taxonomically critical group within *Nymphalidae*, characterized by its remarkable species diversity. Despite its evolutionary significance, the phylogenetic relationships among tribal and subtribal lineages remain poorly resolved. Although mitochondrial genomes have become crucial molecular markers in Lepidoptera phylogenetics, their potential remains underutilized in the systematics of *Satyrinae*. Notably, *Amathusiini* exhibits a particular paucity, with only two congeneric representatives having been comprehensively sequenced to date. **Methods**: We employed high-throughput sequencing to assemble the complete mitochondrial genomes of two *Amathusiini* species, *Discophora sondaica* and *Aemona amathusia*. Our study revealed novel evolutionary insights through comparative genomics, which encompassed all available *Satyrinae* mitochondrial genomes. Additionally, we conducted phylogenetic reconstruction using maximum likelihood and Bayesian inference approaches, utilizing the most extensive dataset to date. **Results**: The closed, circular mitochondrial genomes measure 15,333 bp for *D. sondaica* and 15,423 bp for *A. amathusia*, maintaining the ancestral lepidopteran architecture: 13 protein-coding genes (PCGs), 22 tRNAs, 2 rRNAs, and an AT-rich control region. Comparative analyses of 71 mitochondrial genomes revealed strong evolutionary conservation across multiple parameters: nucleotide composition (AT content range: 77.9–81.8%), codon usage bias (ENC = 30.83–37.55), tRNA secondary structures, and control region organization. All PCGs showed purifying selection signals (Ka/Ks < 1.0), with *atp8* exhibiting the highest evolutionary rate (Ka/Ks = 0.277). Phylogenetic reconstructions yielded congruent tribal-level topologies with strong nodal support: ((*Satyrini* + *Melanitini*) + (*Amathusiini* + *Elymniini*) + *Zetherini*), confirming a sister relationship between *Amathusiini* and *Elymniini*. Within *Satyrini*, five subtribes formed monophyletic groups: *Ypthimina*, *Erebiina*, *Maniolina*, *Satyrina*, and *Melanargiina*, arranged as ((*Ypthimina* + (*Erebiina* + *Maniolina*)) + (*Satyrina* + *Melanargiina*)). *Mycalesina*, *Lethina*, and *Parargina* comprised a well-supported clade (BS = 100%; PP = 1.0), though internal relationships required further resolution due to *Lethina*’s polyphyly. **Conclusions**: This study provides novel insights into mitochondrial genomic evolution within the *Satyrinae* subfamily while elucidating the efficacy of mitogenomic data for resolving deep phylogenetic relationships within this ecologically significant subfamily. Our findings establish critical genome baselines for further systematic research and underscore essential pathways for refining subtribal-level taxonomy through integrative molecular approaches.

## 1. Introduction

Mitochondria are indispensable organelles in eukaryotic cells that serve as central hubs for cellular processes such as ATP production via oxidative phosphorylation, the maintenance of redox balance, the regulation of calcium signaling cascades, and the integration of key metabolic pathways. Unlike nuclear DNA, the mitochondrial genome (mitogenome) represents a semi-autonomous genetic system with unique inheritance patterns and distinct evolutionary dynamics [1]. Insect mitogenomes typically exist as circular DNA molecules (14 to 20 kb) containing 37 canonical genes: 13 protein-coding genes (PCGs) encoding core subunits of respiratory chain complexes, 22 transfer RNA genes (tRNAs), 2 ribosomal RNA genes (rRNAs), and an AT-rich non-coding control region (CR) [2]. Due to their structural simplicity, accelerated mutation rates, predominantly maternal inheritance pattern, and limited recombination, mitogenomes have become powerful molecular tools for evolutionary reconstruction and investigating population genetics [2,3,4,5,6].

*Satyrinae*, formerly known as *Satyridae*, is the most diverse subfamily of the *Nymphalidae* family (Lepidoptera: Papilionoidea), comprising over 2500 species found on all continents except Antarctica [7,8]. In China, there are 364 species across 57 genera, primarily inhabiting southeastern regions such as Yunnan, Guangdong, Hainan, and Taiwan [9]. Adult satyrids are typically small to medium-sized and characterized by their slender body and small head. Their coloration is predominantly gray-brown or dark brown, accentuated with black and white markings. Most individuals exhibit prominent spots or circular stripes on their wings, while a few species demonstrate reduced or entirely absent wing ornamentation [9]. The larvae primarily feed on Poaceae species, with several taxa attaining pest status in rice agroecosystems. Notably, some lineages have evolved to exploit alternative hosts, including ferns and monocotyledonous plants such as palms and bamboos. These butterflies have emerged as valuable model organisms for addressing fundamental questions in terms of ecological adaptation [10], developmental biological patterning [11], and conservation biology [12] due to their interspecific diversity and specialization to their habitat. Consequently, the precise taxonomic delineation and robust phylogenetic reconstruction of satyrid butterflies are crucial for advancing research across these disciplines.

The systematic classification of *Satyrinae* continues to pose significant challenges, driven by extraordinary diversity, limited diagnostic morphological synapomorphies, and incomplete molecular markers across taxa. Historically, taxonomic delineations and infra-subfamilial classifications have been subject to frequent revision, resulting in competing classification systematic frameworks [7,9,13,14]. The advent of high-throughput sequencing technologies has revolutionized phylogenetic approaches, enabling molecular systematics to complement traditional morphological analysis. Growing evidence from integrative taxonomic studies strongly supports *Satyrinae* as a monophyletic subfamily within *Nymphalidae* [15,16,17,18,19,20,21]. Phylogenetic reconstruction consistently recovers the close evolutionary relationship between *Satyrinae* and three nymphalid subfamilies: *Charaxinae*, *Calinaginae*, and *Morphinae* [15,16,17,18,21]. Notably, some classifications propose the conclusion of *Morphini*, *Amathusiini*, and *Brassolini* tribes within *Satyrinae* [19,20]. The current taxonomy recognizes 9 tribes and 16 subtribes in *Satyrinae* [22], with *Satyrini* accounting for approximately 88% of subfamilial diversity through its 13 constituent subgroups. In particular, 12 of these subtribes (excluding *Pronophilina*) exhibit predominant distributions in China [22]. Despite increasing support for *Satyrinae*’s subfamily validity, critical uncertainties persist regarding (1) taxonomic boundaries of major clades, (2) inter-clade phylogenetic relationships, and (3) analytical challenges arising from long-branch attraction artifacts associated with rapid radiative evolution [8,19,20,22,23,24,25]. Marín et al. (2011) [22] established four strongly supported clades for current systematic schemes [8,19,20,25]: (I). *Brassolini* + Morphini, (II). *Elymniini* + *Amathusiini* + *Zetherini* + (*Dirini* + *Melanitini*), (III). *Haeterini*, and (IV). *Satyrini*. Notably, evolutionary relationships among these clades remain unresolved phylogenetic challenges [22]. Subsequently, Yang and Zhang (2015) conducted phylogenetic reconstruction of five Chinese *Satyrinae* tribes (*Satyrini*, *Amathusiini*, *Zetherini*, *Elymniini*, and *Melanitini*) through the combined analysis of two ribosomal genes and four PCGs [23]. These findings confirmed the inclusion of *Amathusiini* within *Satyrinae*, reinforcing previous systematic conclusions. Of particular significance, *Satyrini* was positioned as a long-branched lineage occupying at the basal phylogenetic position, thereby corroborating earlier findings [8,19]. Their phylogenetic analysis revealed tribal relationships as (*Amathusiini* + (*Zetherini* + (*Elymniini* + *Melanitini*))) [23], though this topology conflicts with alternative hypotheses proposed in other studies, including (*Melanitini* + (*Zetherini* + (*Elymniini* + *Amathusiini*))) [8] and ((*Elymniini* + *Melanitini*) + (*Zetherini* + *Amathusiini*)) [20], underscoring the ongoing controversies in *Satyrinae* phylogenetics. It should be noted that existing phylogenetic frameworks have predominantly relied on morphological characteristics and combined partial mitochondrial sequence data, highlighting the necessity for genome-scale datasets to enhance phylogenetic resolution.

The emergence of high-throughput sequencing technologies has revolutionized insect mitochondrial genome sequencing. Nevertheless, mitogenomic coverage within *Satyrinae* remains exceptionally sparse, with *Amathusiini* exhibiting particular paucity; to date, merely two congeneric representatives have been comprehensively sequenced. Recently, several investigations in this subfamily predominantly relied on mitogenomic fragments combined with limited taxonomic representation [26,27,28,29,30,31,32,33,34,35,36]. This current paucity of complete mitogenomes fundamentally impedes the elucidation of evolutionary dynamics governing the mitogenomic architecture and phylogenetic framework within this ecologically significant group. These critical knowledge gaps necessitate large-scale phylogenetic reconstruction using comprehensive mitogenomic datasets with enhanced taxonomic sampling to elucidate both phylogenetic relationships and evolutionary trajectories in *Satyrinae*.

This study presents the complete mitogenome characterization and comparative analysis of two *Amathusiini* species, *Discophora sondaica* and *Aemona amathusia*. Furthermore, we performed the structural reannotation of eight published satyrid mitogenomes originally assembled from publicly available genome resources. To enhance the phylogenetic resolution, we established the most comprehensive mitochondrial phylogenomic framework for *Satyrinae* to date, integrating curated datasets from 71 species spanning all 30 recognized genera within this subfamily for which mitogenome data are currently available. Through this integrated approach combining novel genome characterization, data quality refinement, and expanded taxonomic sampling, our findings reveal unprecedented details of mitogenomic organization patterns, resolve long-standing phylogenetic ambiguities, and establish a robust molecular foundation for reconstructing phylogenetic relationships across major *Satyrinae* lineages.

## 2. Materials and Methods

### 2.1. Sample Collection and Genomic DNA Extraction

Adult specimens of *D. sondaica* and *A. amathusia* were field-collected from JiuFu Mount, Sanming City, Fujian Province, China (118.093 E, 26.103 N) in 2024. Following standard entomological protocol, fresh specimens were immediately preserved in absolute ethanol and stored at −80 °C prior to DNA extraction. Morphological identification was performed using diagnostic characters from established taxonomic keys. All specimens were archived in the Fujian Provincial Key Laboratory of Resources and Environment Monitoring and Sustainable Management and Utilization at Sanming University. Total genomic DNA was extracted from thorax musculature of individually processed specimens using the DNeasy tissue kit following the manufacturer’s protocols (Qiagen, Beijing, China).

### 2.2. Library Preparation and Sequencing

High-molecular-weight DNA (1 μg) was mechanically sheared to approximately 500 bp fragments using a Covaris M220 disruptor and then used to construct short-insert libraries according to the manufacturer’s instructions (TruSeq™ Nano DNA Sample Prep Kit, Illumina, San Diego, CA, USA). Subsequently, high-throughput sequencing was performed on an Illumina NovaSeq 6000 platform (BIOZERON Co., Ltd., Shanghai, China) with a paired-end read length of 150 bp.

### 2.3. Pre-Processing, Read Filtering, Mitogenome Assembly, and Annotation

Raw reads were filtered using Trimmomatic v0.39 [37] to remove reads with adaptors, low quality (Q < 20), ambiguous bases (>10% N-content), and duplicated sequences. A combined assembly strategy was employed for mitogenome reconstruction by integrating de novo assembly with reference-based scaffolding. The workflow comprised three iterative steps: (1) primary assembly using MitoZ v2.3 [38], followed by BLASTn verification against the NCBI Refseq mitogenome database; (2) contig refinement via BLAST v2.8.1+ alignment to curate reference mitogenomes (>80% query coverage) with manually correction based on conserved gene order in Lepidoptera mitochondrial architectures; and (3) topological validation through MUMmer 3.23 whole-genome alignment to confirm circular chromosome continuity and the absence of assembly breaks [39]. Annotation procedures were performed as follows: Structural elements (PCGs, tRNAs, and rRNAs) were identified via the MITOS webserver (http://mitos.bioinf.uni-leipzig.de/index.py, accessed on 16 November 2024) [40] with standard metazoan mitogenome parameters. Gene boundary determination involved Inter reciprocal BLASTp search against the Lepidoptera mitochondrial protein database. Start/stop codon adjustments were performed in SnapGene View through multiple alignment with the reference mitogenome (*Stichophthalma louisa*, KP247523). Final visualization was carried out using the Proksee server (https://proksee.ca/, accessed on 18 November 2024) [41]. tRNAscan-SE 2.0.9 [42] with mitochondrial matrix settings was employed for tRNA secondary structure validation, with structural diagrams rendered in Adobe Illustrator 2021 using conserved tRNA cloverleaf schematics. Control region analysis incorporated Tandem Repeats Finder (http://tandem.bu.edu/trf/trf.html, accessed on 21 November 2024) using the default parameters [43]. The complete circular mitogenomes of *D. sondaica* (PQ845452) and *A. amathusia* (PQ846667) have been deposited in GenBank.

### 2.4. Nucleotide Sequence Composition Analysis

Genome-wide nucleotide distribution and codon usage were systematically analyzed using MEGA 11.0 software with invertebrate mitochondrial genetic code [44]. Relative synonymous codon usage (RSCU) values were computed via Phylosuite 1.2.3 [45]. Strand asymmetry was quantified through AT-skew and GC-skew indices calculated as AT-skew = (A − T)/(A + T) and GC-skew = (G − C)/(G + C) [46]. Nucleotide diversity (Pi) across 13 PCGs was calculated using DNASP 6.0 [47] with a 100 bp sliding window. Nonsynonymous substitution and synonymous substitution ratios (Ka/Ks) were determined using DNASP 6.0. The effective number of codons (ENC) was computed via CodonW 1.4.2 [48] using mitochondrial codon frequency tables.

### 2.5. Phylogenetic Inference

Phylogenetic reconstruction was performed using a comprehensive dataset comprising mitogenomes from 71 *Satyrinae* species representing 30 recognized genera, incorporating two novel sequences generated in this study and 69 publicly available records (Table S1). Three *Charaxinae* species (*Polyura arja*, *Polyura eudamippus*, and *Charaxes kahruba*) served as outgroups for rooting purposes. Mitogenomic features (PCGs and RNAs) were systematically extracted using Phylosuitev1.2.3 [45]. The initial nucleotide alignment of 13 PCGs and RNA components was conducted using MAFFT 7.313 [49]. The nucleotide sequence alignment results for these 13 PCGs were optimized with MACSE 2.03 [50], and ambiguously aligned fragments were removed in batches with Gblocks 0.91b [51] under relax parameters. Gaps in RNA sequences were removed with TrimAl 1.4. rev15 [52]. Following manual verification in MEGA 11.0, a concatenated matrix (13 PCGs + 2 tRNAs + 22 tRNAs) was conducted using Phylosuitev1.2.3. The optimal partitioning model for Bayesian inference (BI) analysis was determined using PartitionFinder2 2.1.1 [53] with a greedy algorithm using the Akaike information criterion (AIC) (Table S2). Bayesian phylogenetic reconstruction was then conducted in MrBayes 3.2.6 [54]. Two parallel runs with four independent Markov chains were executed for 3,000,000 generations, sampling trees every 100 generations. Convergence was confirmed when the average standard deviation of split frequencies fell below 0.01. Following a 25% burn-in removal, the remaining trees from each run were combined to calculate posterior probability (PP) through majority-rule consensus. Model selection for maximum likelihood (ML) analysis was implemented in ModelFinder 2.2.0 [55] using the Bayesian information criterion (BIC) with a distribution-free rate model (Table S3). The ML tree was subsequently constructed using IQ-TREE 1.6.8 [56] with an edge-linked partition model. Nodal bootstrap support (BS) was assessed through 5000 ultrafast bootstrap replicates generated during tree search [57]. All phylogenetic trees were visualized using FigTree v1.4.4 [58].

## 3. Results and Discussion

### 3.1. Mitogenome Organization and Base Composition

**Figure 1:**
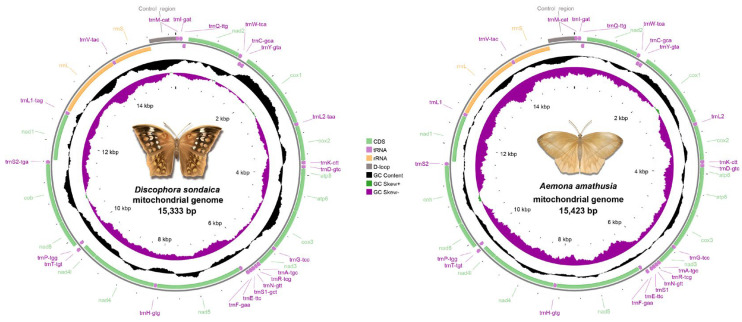
Gene maps for the *Discophora sondaica* and *Aemona amathusia* mitochondrial genomes. Protein-coding genes, ribosomal RNA genes, and transfer RNA genes are shown with standard abbreviations and are marked in green, yellow, and purple, respectively. Arrows indicate the orientation of gene transcription. Pictures of *D. sondaica* and *A. amathusia* were hand-drawn by Xinyue Wang.

Whole-genome sequencing of *D. sondaica* (6254.9 Mb raw data) and *A. amathusia* (5128.9 Mb raw data) was performed using 150 bp paired-end Illumina sequencing. After quality filtering, 6.11 Gb and 4.99 Gb of high-quality reads were obtained for subsequent assembly. De novo assembly generated the complete circular mitogenomes of *D. sondaica* (15,333 bp) and *A. amathusia* (15,423 bp), both exhibiting the characteristic double-stranded structure of insect mitogenomes (Figure 1) [2,26,27,28,29,30,31]. Gene content analysis revealed the conserved repertoire of 37 mitochondrial genes in both species: 13 PCGs, 22 tRNAs, 2 rRNAs (*rrnL* and *rrnS*), and a single non-coding control region. Strand-specific distribution showed 23 genes (9 PCGs and 14 tRNAs) transcribed from the majority strand (J-strand), with the remaining 14 genes (4 PCGs, 2 rRNAs, and 8 tRNAs) located on the minority strand (N-strand) (Table 1). Additionally, the mitogenomic organization of eight previously sequenced satyrid species was reannotated, and the results are presented in Table S4. Comparative analysis with other reported Satyrid mitogenomes revealed remarkable size across the subfamily, with the total lengths ranging from 15,054 bp (*Coenonympha glycerion*) to 16,129 bp (*Bicyclus anynana*) (Figure S1). Obviously, all analyzed mitogenomes maintain identical gene order and transcriptional polarity to previously reported architectures [26,27,28,29,30,31,32,33,34,35,36,59,60,61,62,63,64,65,66,67,68,69,70,71,72,73]. While the 13 PCGs exhibit conserved size, length polymorphisms primarily occur in non-coding regions, particularly the control region (Figure S1).

**Figure 2:**
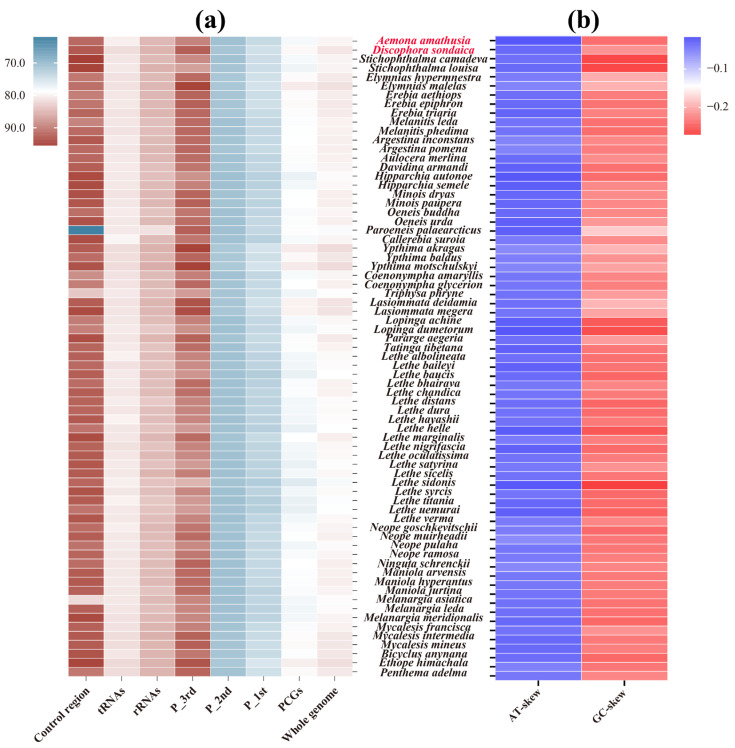
Nucleotide compositions of the satyrid mitochondrial genomes. (**a**) A + T content of the whole genome, protein-coding genes (PCGs), first codon position of the PCGs (P_1st), second codon position of the PCGs (P_2nd), third codon position of the PCGs (P_3rd), ribosomal RNA genes (rRNAs), transfer RNA genes (tRNAs), and the control region; (**b**) AT- and GC-skew of the whole genome. Species with mitochondrial genomes sequenced in this study are marked in red.

The mitogenomes of *D. sondaica* and *A. amathusia* exhibit a strong AT nucleotide bias with total AT content of 80.9% and 79.6%, respectively. This composition bias was observed across all functional elements (13 PCGs, 22 tRNAs, 2 rRNAs) within these newly sequenced mitogenomes (Table 2), mirroring the compositional patterns of other satyrid mitogenomes (Figure 2a). Hierarchical analysis revealed the control region as the most AT-rich element (93.3% and 91.9%), followed by rRNA genes (85.3% and 84.9%), tRNA clusters (81.7% and 80.2%), and 13 PCGs (79.3% and 77.8%). This hierarchical AT distribution aligns with most reported satyrid mitogenomes except *Paroeneis palaearcticus*, which displays an anomalously low AT content of the control region (61.8%) [28]. At the codon level, the third position in PCGs maintains significantly higher AT content (85.3–95.4%) compared to the first (71.7–75.3%) and second positions (70.1–71.3%) across all analyzed species (Figure 2a). Comparative analysis of five *Satyriane* tribes revealed limited compositional variation, with whole-mitogenome AT contents ranging narrowly from 79.7% in *Satyrini* to 81.1% in *Zetherini* (Figure S2). Nucleotide skew analysis revealed conserved patterns: AT-skew values range from −0.055 (*Neope muirheadii*) [26] to −0.015 (*A. amathusia*), while GC-skew values vary between −0.273 (*Lethe sidonis*) and −0.179 (*P. palaearcticus*) [28] (Table 2, Figure 2b). These results corroborate the proposed standard insect mitogenome skewness profiles [46,74], suggesting the evolutionary conservation of replication–strand mutation biases in Lepidoptera.

### 3.2. Protein-Coding Genes and Codon Usage

In both newly sequenced satyrid mitogenomes, most PCGs exhibited canonical ATN (N = A/T/G/C) initiation codons. A notable exception was observed in *cox1*, which uniquely employed CGA as its start codon (Table 1). This unconventional use of CGA as an initiation codon for *cox1* represents a conserved feature consistently documented across *Satyrinae* mitogenomes [26,27,28,29,30,31,32,33,34,35,36,59,60,61,62,63,64,65,66,67,68,69,70,71,72,73]. Regarding termination signals, complete TAA stop codons were predominant among PCGs in both mitogenomes. However, six genes (*cox1*, *cox2*, *nad1*, *nad4*, *nad5*, and *cob*) displayed incomplete termination signals consisting of a single T residue (Table 1). Such truncated stop codons are commonly observed in insect mitogenomes and are hypothesized to be completed through post-transcriptional polyadenylation during mRNA maturation process [75].

**Figure 3:**
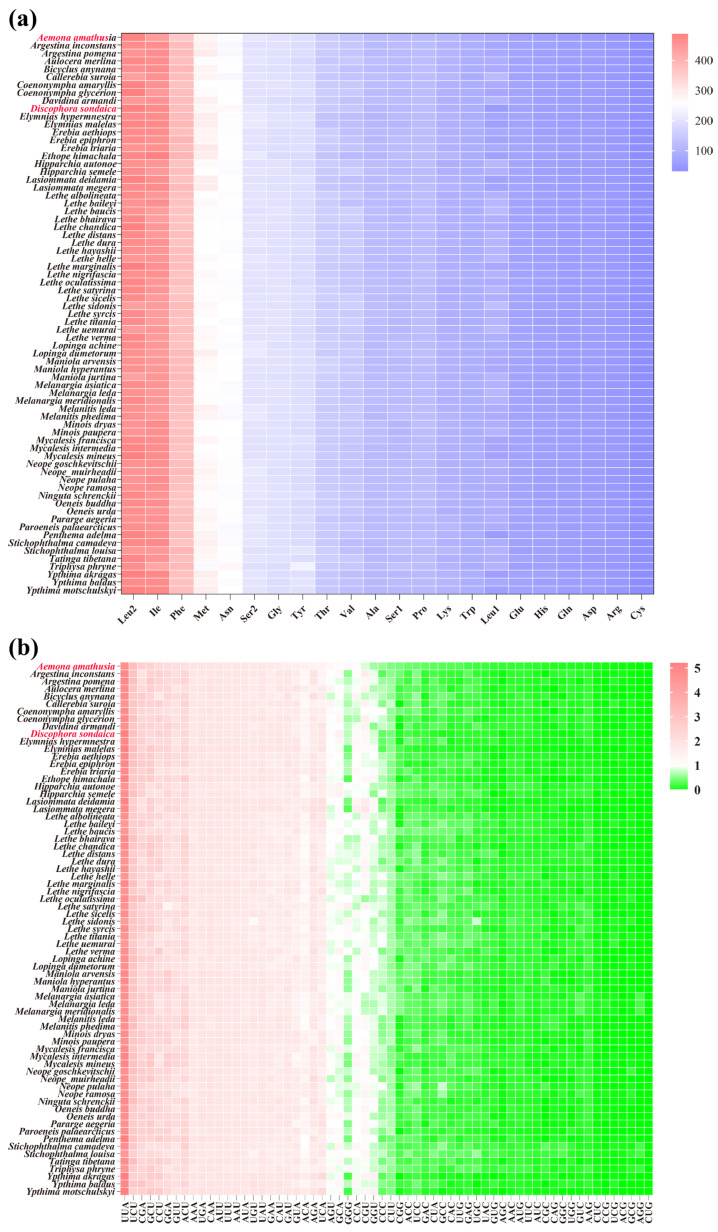
Comparison of codon usage of the 71 selected *Satyrinae* mitochondrial genomes. (**a**) Composition of amino acids; (**b**) relative synonymous codon usage (RSCU). Species with mitochondrial genomes sequenced in this study are marked in red. Codon families are provided on the *x*-axis.

The codon family composition and RSCU patterns of the 13 PCGs were analyzed for the two newly sequenced mitogenomes. To minimize potential biases arising from incomplete stop codons, all stop codons were excluded from the analysis. The combined length of the 13 PCGs was 11,202 bp in *D. sondaica* and 11,196 bp in *A. amathusia*, encoding 3727 and 3722 amino acids (AAs). These values align with those reported for other *Satyrinae* mitogenomes, which range from 3682 AAs in *Mycalesis intermedia* [64] to 3755 AAs in *Tatinga tibetana* (Figure S3). Among the encoded amino acids, Leu2, Ile, Phe, Met, and Asn were the most abundant in both species, whereas Cys exhibited the lowest frequency (Figure S4). This distribution mirrors trends observed across all available *Satyrinae* mitogenomes (Figure 3a). RSCU analysis showed a pronounced bias toward codons ending with A/U at the third position values in both newly sequenced species (Figure S5). The eight most prevalent codons shared by both mitogenomes were UUA, UCU, CGA, GCU, CCU, GGA, GUU, and ACU. Notably, this codon preference pattern is conserved in the PCGs of most other *Satyrinae* species (Figure 3b). Distinct absences were observed in specific codons: three codons (CUG, CCG, and AGG) were entirely absent in *D. sondaica*, while two (CUG and AGG) were missing in *A. amathusia*. Furthermore, GC-rich codons showed markedly reduced usage across most satyrid species (Figure 3b). For instance, *Neope goschkevitschii* lacks only one codon (CCG) [67], whereas six codons (AGG, CUG, GCG, ACG, CCG, and AGC) are absent in *Ypthima motschulskyi* [26] (Figure 3b). These findings are consistent with broader lepidopteran codon usage trends [21,76], reinforcing the strong correlation between genomic GC content and codon preference evolution [77,78].

**Figure 4:**
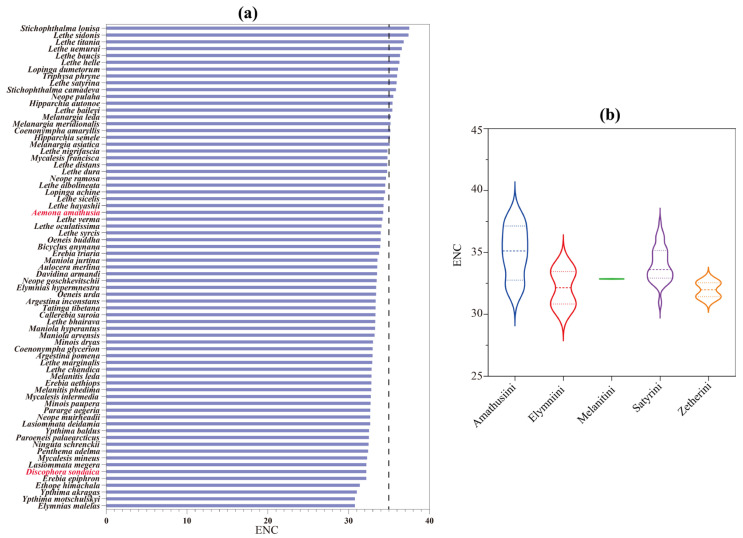
The effective number of codons (ENC) of *Satyrinae* mitochondrial genomes. (**a**) The ENC of 71 selected *Satyrinae* species; (**b**) the average ENC of five satyrid tribes. Species with mitochondrial genomes sequenced in this study are marked in red. The three dashed lines on the violin plots from top to bottom are third quartile, median, and first quartile, respectively.

The ENC shows an inverse relationship with codon usage bias intensity, typically ranging between 20 and 61 in biological systems. Using an established critical threshold (ENC = 35) for assessing codon selection patterns [79], our analysis revealed distinct preferences across studied species. As shown in Figure 4a, *D. sondaica* (ENC = 32.23) and *A. amathusia* (ENC = 34.34) exhibited a stronger codon preference, falling below this benchmark. Comparative analysis of available satyrid mitogenomes identified a continuum of ENC values from 30.83 in *Elymnias malelas* and *Y. motschulskyi* [26] to 37.55 in *S. louisa*. Tribal-level comparisons within *Satyrinae* showed significant variation: *Amathusiini* exhibited the highest average ENC (35.01), followed sequentially by *Satyrini* (34.01), *Melanitini* (32.86), and *Elymniini* (32.15), while *Zetherini* recorded the lowest average (31.99) (Figure 4b). This systematic evaluation confirms universal but variable codon usage bias across all examined satyrid mitogenomes.

### 3.3. Transfer RNA, Ribosomal RNA Genes, and Control Region

Comparative analysis of the two mitogenomes revealed 22 typical tRNAs in both *D. sondaica* and *A. amathusia*, exhibiting size variation from 61 bp (*trnS1*) to 71 bp (*trnK*) (Table 1). Strand distribution analysis showed eight tRNAs (*trnQ*, *trnC*, *trnY*, *trnH*, *trnP*, *trnL1*, *trnV*, and *trnF*) encoded on the N-strand, with the remaining fourteen positioned on the J-strand. Secondary structural predictions demonstrated that all tRNAs adopted the typical clover-leaf conformation except *trnS1*, which displayed a reduced dihydrouridine (DHU) arm forming a simple loop structure (Figures S6 and S7). The DHU stem truncation in *trnS1* represents a conserved architectural feature observed across in insect mitogenomes, including all available satyrid species sequences [26,27,28,29,30,31,32,33,34,35,36,59,60,61,62,63,64,65,66,67,68,69,70,71,72,73]. Notably, G-U wobble pairs and U-U mismatches were detected at frequencies of 24 and 27 instances in *D. sondaica* and *A. amathusia*, respectively (Figures S6 and S7). Quantitative analysis revealed a predominance of G-U mismatches over the noncanonical pair types in both species, a distribution pattern consistent with previous reports of tRNA structural features in insect mitogenomes [80,81,82,83].

Consistent with other *Satyrinae* mitogenomes, both rRNA genes (*rrnL* and *rrnS*) in *D. sondaica* and *A. amathusia* exhibit N-strand encoding. These rRNA genes maintain conserved spatial organization within the mitogenome, flanking *trnV* while occupying their characteristic position between *trnL1* and the control region [26,27,28,29,30,31,32,33,34,35,36,59,60,61,62,63,64,65,66,67,68,69,70,71,72,73]. Comparative analysis reveals distinct size variations: *D. sondaica* possesses *rrnL* (1331 bp) and *rrnS* (770 bp), while *A. amathusia* demonstrates large dimensions with *rrnL* (1399 bp) and slightly reduced *rrnS* (767 bp). Notably, *A. amathusia*’s *rrnL* represents the longest recorded among sequenced satyrid species, exceeding *Mycalesis mineus* minimum length (1011 bp) by 388 base pairs [63] (Figure S1). The small subunit rRNA (*rrnS*) measurement in both novel mitogenomes aligns with subfamily standards, falling within established parameters for this subfamily ranging from 546 bp in *Ypthima akragas* [32] to 852 bp in *P. palaearcticus* [28] (Figure S1).

Characterized as both the largest intergenic spacer and primary replication initiation site in insect mitogenomes, the control region has been successfully identified in our two newly sequenced mitogenomes. This non-coding segment is consistently located between the *rrnS* and *trnM* genes (Figure 1 and Table 2). Comparative analysis reveals size variations ranging from 181 bp (*C. glycerion*) to 1322 bp (*B. anynana*), with significant AT content fluctuations from 61.8% (*P. palaearcticus*) [28] to 95.6% (*Stichophthalma camadeva*). Our findings demonstrate that these length polymorphisms account for most of the observed mitogenome size variation among species (Figure S1). Additionally, the control regions of the two newly sequenced mitogenomes exhibited an absence of tandem repeat elements (>50 bp), a genome feature commonly observed in lepidopteran insects [84]. However, comparative analysis revealed the preservation of three hallmark structural elements characteristic of lepidopterans across all three mitogenomes: (1) a conserved “ATAGA” motif adjacent to *rrnS* followed by a poly-T stretch, corresponding to the minority strand replication origin; (2) microsatellite-like elements (TA)n or (AT)n initiated by an “ATTTA” signature sequence; and (3) a distinct Ploy (A) tract preceding *trnM* (Figure S8).

### 3.4. Nucleotide Diversity and Evolutionary Rate Among Satyrinae Mitogenomes

**Figure 5:**
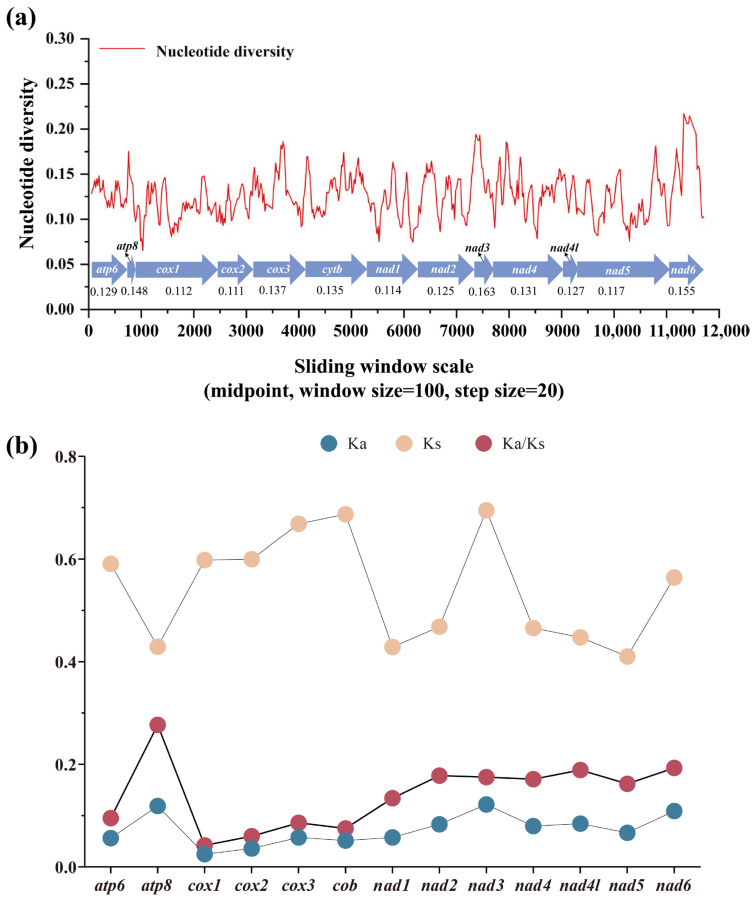
Gene variation in 13 PCGs based on 71 satyrid species. (**a**) The sliding-window analysis shows the value of nucleotide diversity; (**b**) the Ka, Ks, and Ka/Ks of each PCG among *Satyrinae* representatives. Ka, the number of nonsynonymous substitutions per nonsynonymous site; Ks, the number of synonymous substitutions per synonymous site.

Nucleotide diversity analysis serves as a critical tool for detecting genome regions with elevated sequence divergence, providing a valuable guideline for developing species- or group-specific molecular markers. This approach proves particularly advantageous in evolutionary studies of morphological conserved taxa [85,86]. Through sliding-window analysis of aligned PCGs, we quantified nucleotide variation patterns across all *Satyrinae* mitogenomes using Pi values. Our results revealed considerable variation in nucleotide diversity among the 13 PCGs, with average Pi values spanning from 0.111 (*cox2*) to 0.163 (*nad3*) (Figure 5a). Furthermore, *nad3* emerged as the most variable gene (Pi = 0.163), closely followed by *nad6* (Pi = 0.155) and *atp8* (Pi = 0.141). In contrast, *cox2* (Pi = 0.111) and *cox1* (Pi = 0.112) displayed relatively conserved evolutionary patterns, indicating the lowest nucleotide diversity among examined PCGs. These findings align with our previous report on satyrid mitogenome evolution [31] yet present an intriguing contrast to the divergence patterns observed in other butterfly groups. *nad6* was identified as a fast-evolving gene in *Papilionidae* [87] and *Limenitidinae* [88]. Our findings highlight that genes exhibiting an accelerated divergence rate may serve as reliable DNA barcodes for both population genetics investigations and species identification in *Satyrinae*.

The Ka/Ks ratio (ω) serves as a crucial metric for assessing selective pressures on protein-coding genes, distinguishing between positive selection (ω ˃ 1), neutral evolution (ω = 1), and purifying selection (0 < ω < 1) [89]. We calculated ω values for all 13 PCGs across all available *Satyrinae* mitogenomes (Figure 5b) to evaluate evolutionary rate variation among mitochondrial genes. Our analysis demonstrated that all 13 PCGs exhibited ω values significantly below unity (ω < 1), which is consistent with previous reports in satyrid butterflies [26,31], thereby confirming their predominant evolution under purifying selection conditions. This evolutionary conservation supports the utility of these PCGs for phylogenetic reconstruction within *Satyrinae*. Notable variation emerged in selected intensity across individual genes; the *atp8* gene exhibited the highest evolutionary rate (ω = 0.277), followed by *nad6* (ω = 0.193) and *nad4l* (ω = 0.189). In contrast, the *cox1* gene displayed exceptional conservation with the lowest ω value (0.042). These findings indicate that *atp8* experiences relatively relaxed selective constraints compared to other mitochondrial genes, while *cox1* undergoes the strongest purifying selection within *Satyrinae* mitogenomes. The observed hierarchy of evolutionary rates aligns with documented patterns in other Lepidoptera lineages, including *Papilionidae* [87] and *Limenitidinae* [88], implying conserved mechanisms of mitochondrial genome evolution across butterfly taxa.

### 3.5. Phylogenetic Analysis

**Figure 6:**
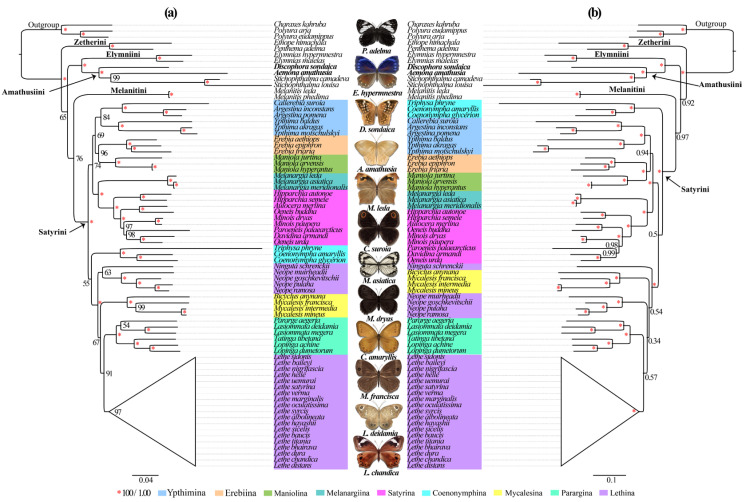
Phylogenetic trees inferred from maximum likelihood (**a**) and Bayesian inference (**b**) method based on the dataset (13 PCGs + 2 tRNAs + 22 tRNAs). Numbers on a node represent the bootstrap support and posterior probability. The species with mitogenomes sequenced in this study are marked in bold. The satyrid butterfly pictures in the middle were hand-drawn by Xinyue Wang.

The *Satyrinae* subfamily represents a remarkably diverse lineage of exceptional species richness, serving as model organisms in evolutionary studies [10,11,12]. Despite its biological significance, the current phylogenetic understanding of its tribal and subtribal relationships remains constrained by inadequate sampling and insufficient molecular data. To address these limitations, we conducted an extensive phylogenetic analysis incorporating expanded mitochondrial genomic data (13 PCGs + 2 rRNAs + 22 tRNAs) and enhanced taxonomic representation across 71 species. Our dataset includes two newly sequenced *Satyrinae* species, collectively representing all 30 mitogenome-available genera within the subfamily. Phylogenetic reconstruction using ML and BI approaches yielded congruent topologies at the tribal levels. Both analyses strongly supported the monophyly of Satyriane (BS = 100%; PP = 1.0) (Figure 6), with five distinct tribal lineages resolved: *Satyrini*, *Amathusiini*, *Zetherini*, *Melanitini*, and *Elymniini*. The reconstructed relationships followed a ((*Satyrini* + *Melanitini*) + (*Amathusiini* + *Elymniini*) + *Zetherini*) configuration across both analytical methods. Notably, *Zetherini* occupied a basal position relative to other tribes, a finding consistent with our previous investigations [31] though conflicting with alternative topologies derived from different datasets that position *Satyrini* as the basal lineage [2,15,19]. Within this framework, *D. sondaica* and *A. amathusia* clustered securely within *Amathusiini* (BS = 100%; PP = 1.0), forming a sister group with *Elymniini*. This configuration aligns with previous mitogenomic analyses [26,29,30,31] and the BI results of Peña and Wahlberg [8], contrasting with earlier morphological assessments suggesting closer affinity between *Elymniini* and *Melanitini* [20,23]. These persistent discrepancies highlight the need for expanded mitogenomic sampling to resolve deep phylogenetic relationships within *Satyrinae*.

Despite the limited taxon sampling within *Satyrini*, both ML and BI analyses consistently supported the monophyly of all analyzed subtribes except for *Lethina*. Within *Lethina*, the genus *Lethe* emerges as phylogenetically distinct. Moreover, the phylogenetic placement of *Ninguta* exhibited incongruence between ML and BI topologies. Our results strongly support *Melanargiina* as a sister to *Satyrina* (BS = 100%; PP = 1.0), corroborating previous findings [26,28,31,36]. The analyses revealed *Ypthimina* as a sister to the *Erebiina* + *Maniolina* clade with moderate to strong support (BS = 69%; PP = 0.94), consistent with our previous mitogenomic study [31]. A notable discordance emerged regarding *Coenonymphina*’s placement: while BI analysis robustly positioned it as a sister to the remaining *Satyrini* subtribes (PP = 1.0), ML analysis indicated an alternative affiliation with the *Mycalesina* + Lethin + *Parargina* clade, albeit with weak nodal support (BS = 55%), mirroring a previous study [25]. We confirmed the closed evolutionary relationships among *Mycalesina*, *Lethina*, and *Parargina* subtribes, aligning with both prior mitogenomic [26,28,29,31,36] and multi-locus studies [8,20,24,25]. However, these inter-subtribal nodes were generally weakly statistically supported across our analyses. This uncertainty likely reflects insufficient taxonomic representations—only 9 of 13 recognized subtribes were included, omitting critical groups like *Eritina*, *Euptychiina*, *Ragadiina*, and *Pronophilin*. Our findings underscore the necessity of expanded global sampling to resolve basal relationships within *Satyrini*. Further studies should prioritize the inclusion of underrepresented subtribes to strengthen phylogenetic inferences and clarify systematic uncertainties.

## 4. Conclusions

In this study, we successfully sequenced and annotated the complete mitogenomes of *D. sondaica* and *A. amathusia*. The two novel mitogenomes exhibited the canonical Lepidoptera gene architecture, maintaining conserved gene orientation and organization patterns observed across the *Satyrinae* subfamily. The estimation of evolutionary rates indicated that universal purifying selection pressures are acting on 13 PCGs. Phylogenetic reconstruction clarified tribal relationships within *Satyrinae*, yielding a topology of ((*Satyrini* + *Melanitini*) + (*Amathusiini* + *Elymniini*) + *Zetherini*), with strong nodal support confirming the sister relationship between *Amathusiini* and *Elymniini*. At the subtribe level within *Satyrini*, we resolved the hierarchy as ((*Ypthimina* + (*Erebiina* + *Maniolina*)) + (*Satyrina* + *Melanargiina*)) while identifying a potential clade comprising *Mycalesina*, *Lethina*, and *Parargina*. However, these relationships require further validation due to moderate bootstrap support. Our findings provide critical mitogenomic resources and a phylogenetic framework for *Satyrinae*, establishing essential groundwork for future studies on the evolutionary dynamics, phylogenetic resolution, genetic diversity, and conservation of this ecologically significant butterfly subfamily.

## Figures and Tables

**Table 1:** The mitogenomic organization of *Discophora sondaica* (DS) and *Aemona amathusia* (AA).

Gene	Coding Strand	Position		Size (bp)		IGN		Start Codon		Stop Codon	
		DS	AA	DS	AA	DS	AA	DS	AA	DS	AA
*trnM*	J	1–69	1–66	69	66	0	0				
*trnI*	J	70–136	69–133	67	65	0	2				
*trnQ*	N	134–201	131–199	68	69	−3	−3				
*nad2*	J	248–1261	245–1258	1014	1014	46	45	ATT	ATC	TAA	TAA
*trnW*	J	1260–1327	1257–1323	68	67	−2	−2				
*trnC*	N	1320–1383	1316–1383	64	68	−8	−8				
*trnY*	N	1384–1450	1384–1449	67	66	0	0				
*cox1*	J	1453–2983	1471–3001	1531	1531	2	21	CGA	CGA	T	T
*trnL2*	J	2984–3051	3002–3068	68	67	0	0				
*cox2*	J	3052–3727	3070–3742	676	673	0	1	ATG	ATG	T	T
*trnK*	J	3728–3798	3743–3813	71	71	0	0				
*trnD*	J	3810–3874	3816–3880	65	65	11	2				
*atp8*	J	3875–4039	3881–4039	165	159	0	0	ATC	ATG	TAA	TAA
*atp6*	J	4033–4710	4033–4710	678	678	−7	−7	ATG	ATG	TAA	TAA
*cox3*	J	4710–5498	4710–5498	789	789	−1	−1	ATG	ATG	TAA	TAA
*trnG*	J	5501–5567	5501–5566	67	66	2	2				
*nad3*	J	5568–5915	5564–5914	348	351	0	−3	ATT	ATA	TAA	TAA
*trnA*	J	5919–5988	5916–5979	70	64	3	1				
*trnR*	J	5988–6049	5979–6040	62	62	−1	−1				
*trnN*	J	6051–6119	6041–6106	69	66	1	0				
*trnS1*	J	6117–6177	6104–6164	61	61	−3	−3				
*trnE*	J	6178–6242	6194–6257	65	64	0	29				
*trnF*	N	6241–6306	6262–6327	66	66	−2	4				
*nad5*	N	6307–8044	6333–8072	1738	1740	0	5	ATT	ATT	T	TAA
*trnH*	N	8045–8108	8073–8137	64	65	0	0				
*nad4*	N	8109–9447	8138–9476	1339	1339	0	0	ATG	ATG	T	T
*nad4l*	N	9449–9739	9477–9764	291	288	1	0	ATG	ATG	TAA	TAA
*trnT*	J	9742–9806	9767–9830	65	64	2	2				
*trnP*	N	9807–9871	9831–9895	65	65	0	0				
*nad6*	J	9874–10404	9901–10428	531	528	2	5	ATT	ATA	TAA	TAA
*cob*	J	10404–11556	10432–11586	1153	1155	−1	3	ATG	ATG	T	TAA
*trnS2*	J	11557–11622	11586–11650	66	65	0	−1				
*nad1*	N	11625–12579	11656–12609	955	954	2	5	ATG	ATA	T	TAA
*trnL1*	N	12580–12648	12613–12681	69	69	0	3				
*rrnL*	N	12663–13993	12684–14082	1331	1399	14	2				
*trnV*	N	13994–14057	14083–14148	64	66	0	0				
*rrnS*	N	14058–14827	14149–14915	770	767	0	0				
Control region		14828–15333	14916–15423	506	508	0	0				

Note: J and N indicate the majority coding strand and the minority strand, respectively; IGN values represent intergenic nucleotides and overlapping nucleotides (−); T indicates the incomplete stop codon.

**Table 2:** Nucleotide composition of *Discophora sondaica* (DS) and *Aemona amathusia* (AA) mitogenome.

Feature	Proportion of Nucleotides (%)										AT-Skew		GC-Skew	
	T		C		A		G		A + T					
	DS	AA	DS	AA	DS	AA	DS	AA	DS	AA	DS	AA	DS	AA
Whole-genome	41.6	40.4	11.6	12.7	39.3	39.2	7.5	7.8	80.9	79.6	−0.028	−0.015	−0.215	−0.239
PCGs	45.9	44.7	10.1	10.9	33.4	33.1	10.6	11.2	79.3	77.8	−0.157	−0.149	0.027	0.013
1st	37.5	37.1	9.8	10.5	37.0	36.5	15.7	16.0	74.5	73.6	−0.006	−0.008	0.232	0.208
2nd	48.6	47.8	16.0	16.2	22.2	22.4	13.3	13.5	70.8	70.2	−0.373	−0.362	−0.092	−0.092
3rd	51.6	49.2	4.4	6.1	41.2	40.4	2.8	4.2	92.8	89.6	−0.113	−0.098	−0.215	−0.184
*rrnL*	39.4	39.7	5.0	4.8	45.5	44.9	10.1	10.6	84.9	84.6	0.072	0.061	0.338	0.377
*rrnS*	39.6	39.8	4.4	5.1	46.4	45.5	9.6	9.6	86.0	85.3	0.079	0.067	0.371	0.306
tRNAs	40.2	39.6	7.6	8.4	41.5	40.6	10.8	11.4	81.7	80.2	0.016	0.012	0.174	0.152
Control-region	51.2	48.2	3.8	5.5	42.1	43.7	3.0	2.6	93.3	91.9	−0.098	−0.049	−0.118	−0.358

## Data Availability

The genome sequence data that support the findings of this study are openly available in GenBank of NCBI at (https://www.ncbi.nlm.nih.gov/, accessed on 31 January 2025) under the accession nos. PQ845452 (*Discophora sondaica*) and PQ846667 (*Aemona amathusia*). The associated BioProject, SRA, and Bio-Sample numbers for *D. sondaica* and *A. amathusia* are PRJNA1231788 and PRJNA1231789, SRR32627473 and SRR32581916, and SAMN47222378 and SAMN47221793, respectively.

## Supplementary Materials

The following supporting information can be downloaded at https://www.mdpi.com/article/10.3390/genes16040447/s1, Figure S1: Comparison of gene lengths for 71 selected *Satyrinae* mitochondrial genomes. Species with mitochondrial genomes sequenced in this study are marked in red; Figure S2: The averaged A + T content of the mitochondrial genomes of five satyrid tribes; Figure S3: Comparison of the number of amino acids for 71 selected *Satyrinae* mitochondrial genomes. Species with mitochondrial genomes sequenced in this study are marked in red; Figure S4: Composition of protein-coding gene amino acids in two newly sequenced mitochondrial genomes; Figure S5: Relative synonymous codon usage (RSCU) patterns in two newly sequenced mitochondrial genomes; Figure S6: Predicted secondary cloverleaf structures for the tRNAs of the *Discophora sondaica*; Figure S7: Predicted secondary cloverleaf structures for the tRNAs of the *Aemona amathusia*; Figure S8: Control region features of in two newly sequenced mitochondrial genomes; Table S1: List of taxa used for the phylogenetic analyses in this study; Table S2: The partitioning schemes and corresponding substitution models determined by PartitionFinder for the concatenated matrix (13 PCGs + 2 tRNAs + 22 tRNAs); Table S3: The partitioning schemes and corresponding substitution models determined by ModelFinder for the concatenated matrix (13 PCGs + 2 tRNAs + 22 tRNAs); Table S4: Reannotation and comparison of mitochondrial genome organizations of 8 previous sequenced satyrid species.
