# Supplementary figures and images for: Complete Mitochondrial Genome of Two Amathusiini Species (Lepidoideae: Nymphalidae: Satyrinae): Characterization, Comparative Analyses, and Phylogenetic Implications
Source: Genes (Basel). 2025 Apr 12;16(4):447. doi: 10.3390/genes16040447 (PMC12026527; doi:10.3390/genes16040447)

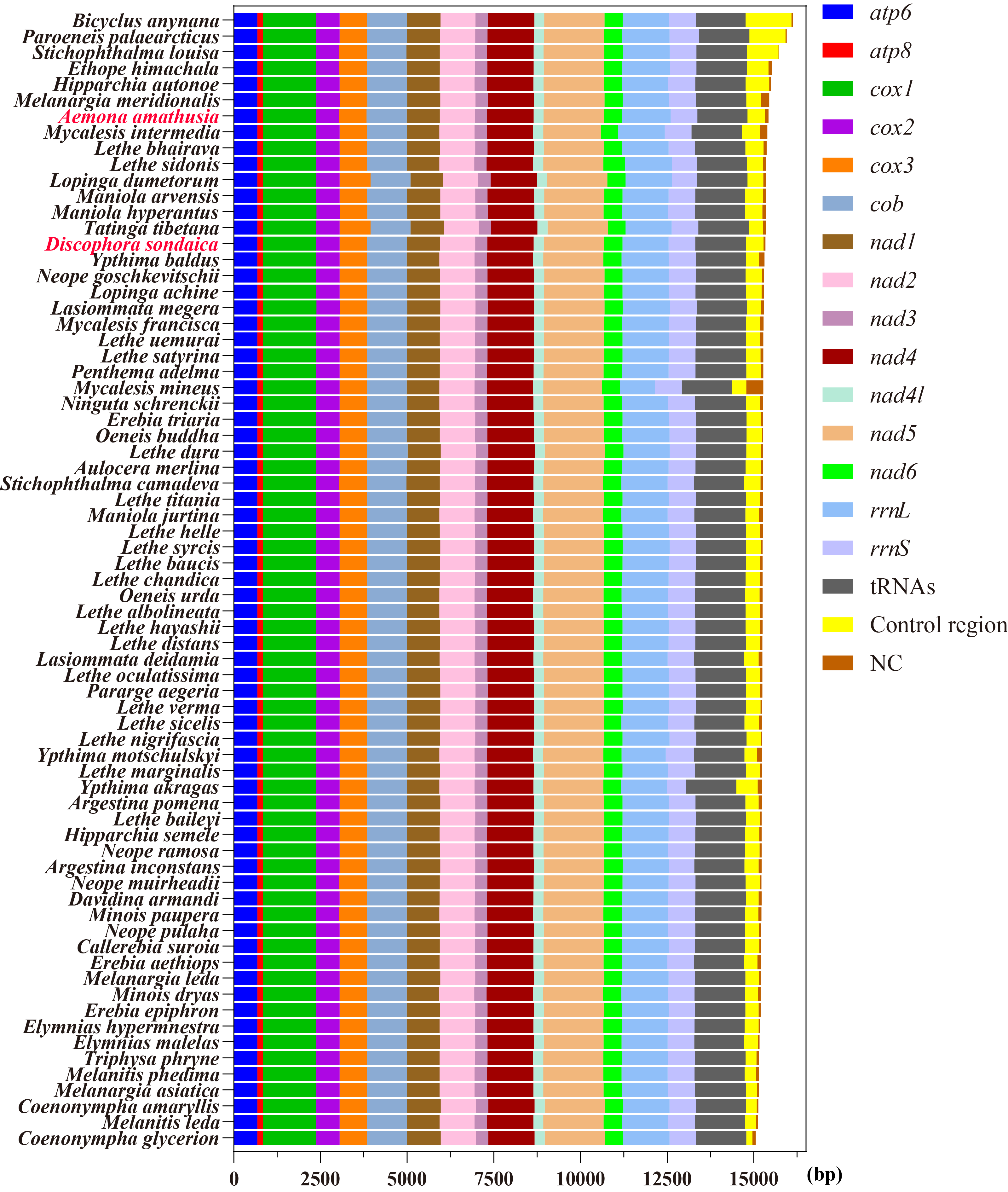

Supplement: Supplementary file 1 [file genes-16-00447-s001.zip › Figure S1.png]

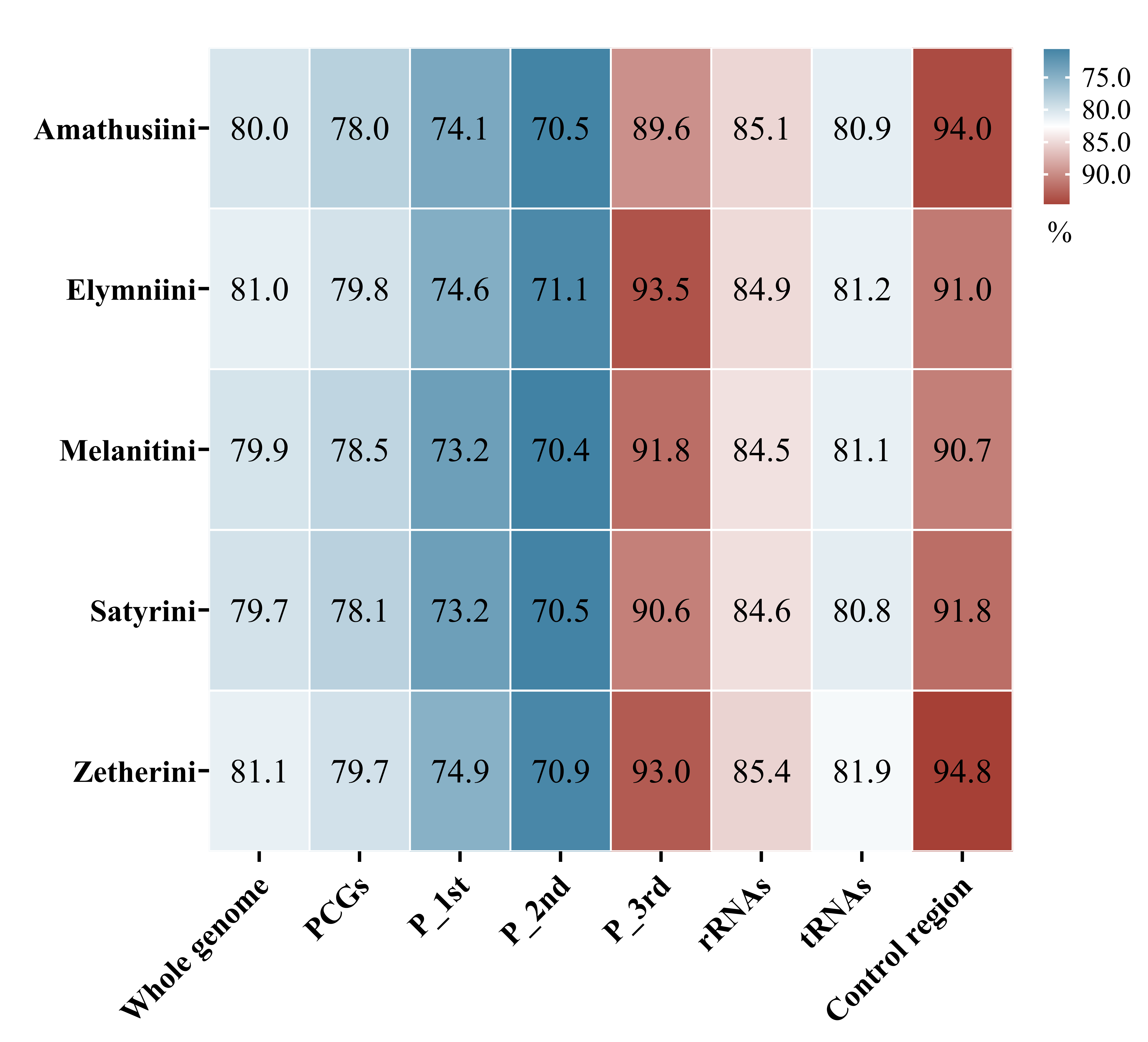

Supplement: Supplementary file 1 [file genes-16-00447-s001.zip › Figure S2.png]

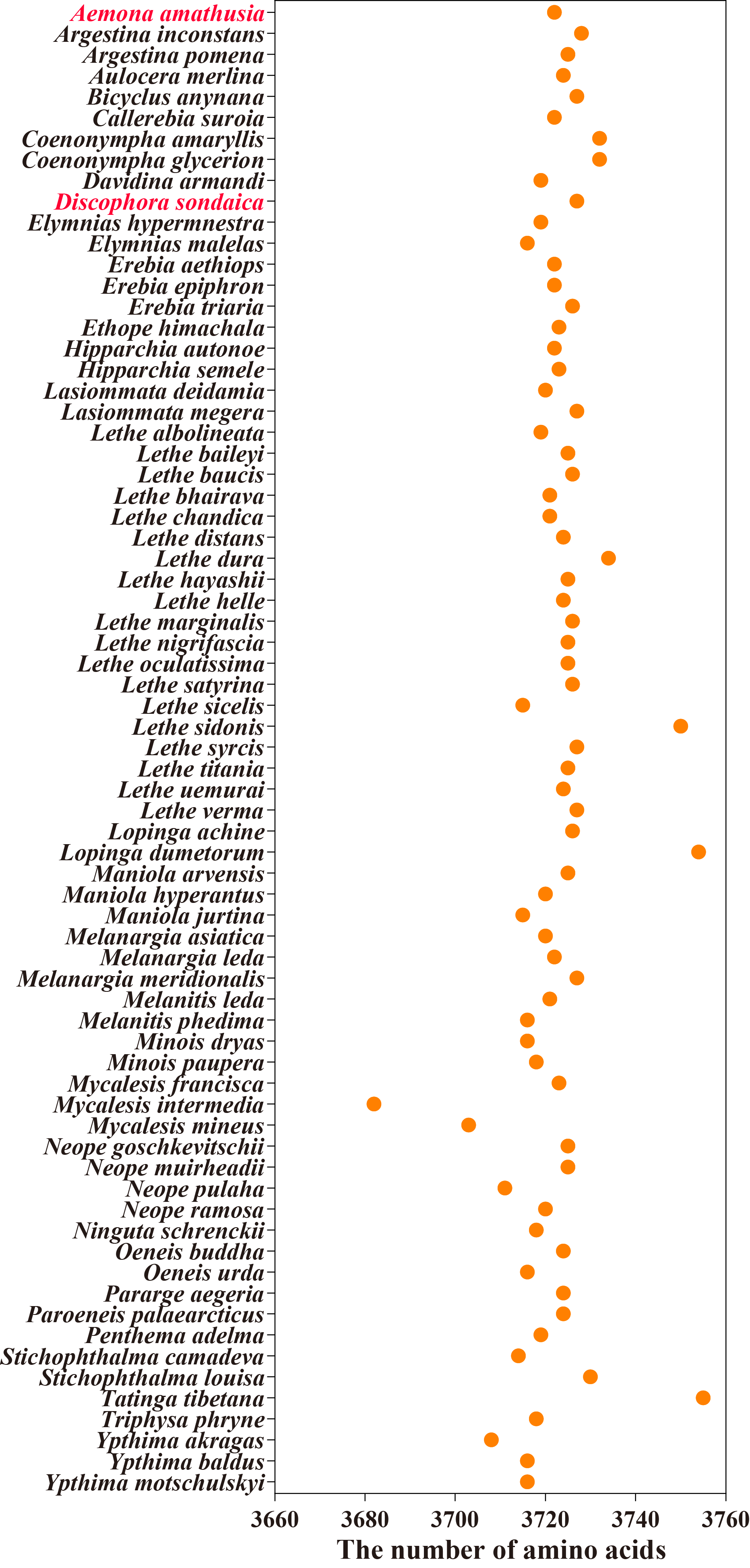

Supplement: Supplementary file 1 [file genes-16-00447-s001.zip › Figure S3.png]

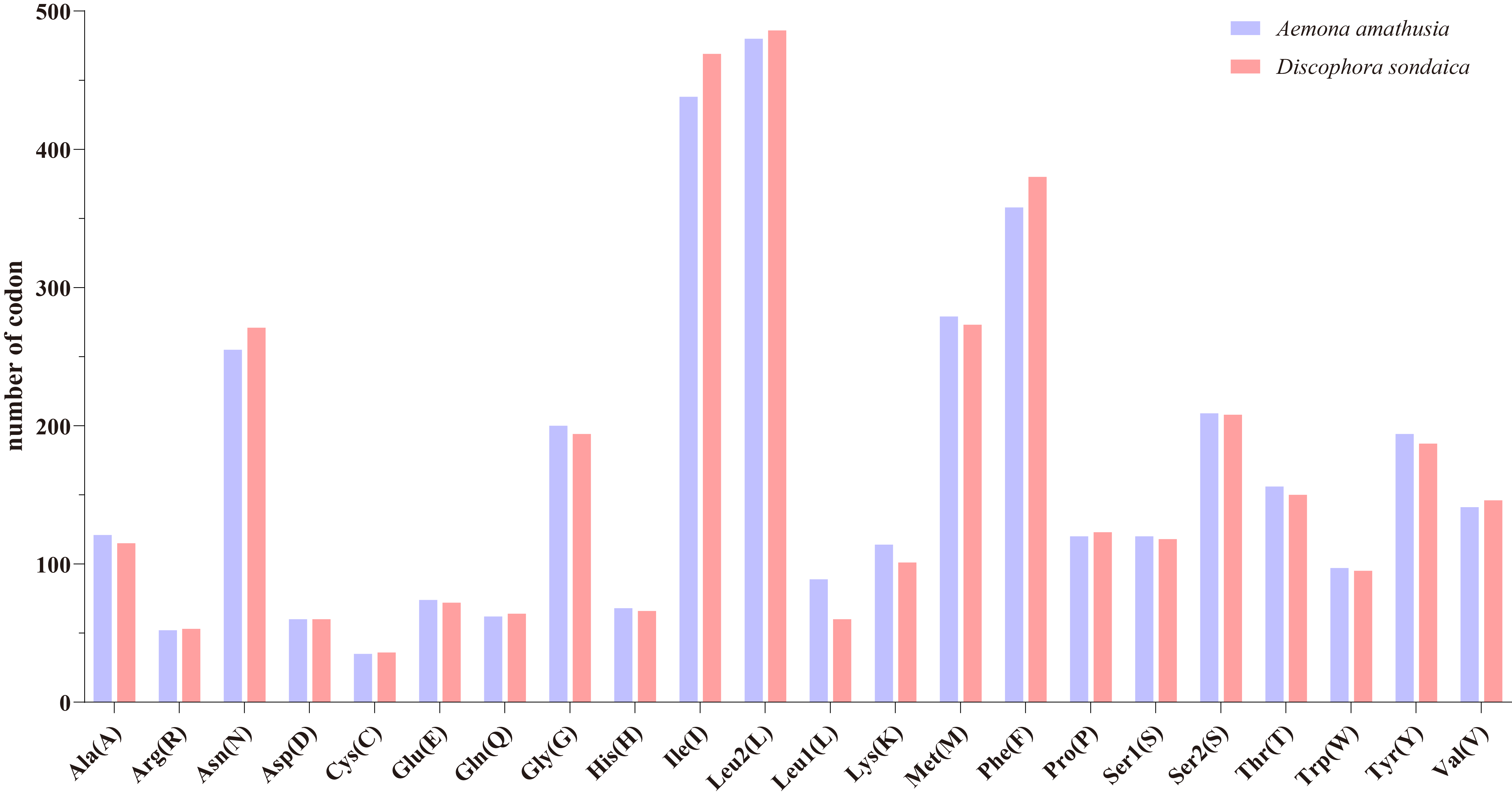

Supplement: Supplementary file 1 [file genes-16-00447-s001.zip › Figure S4.png]

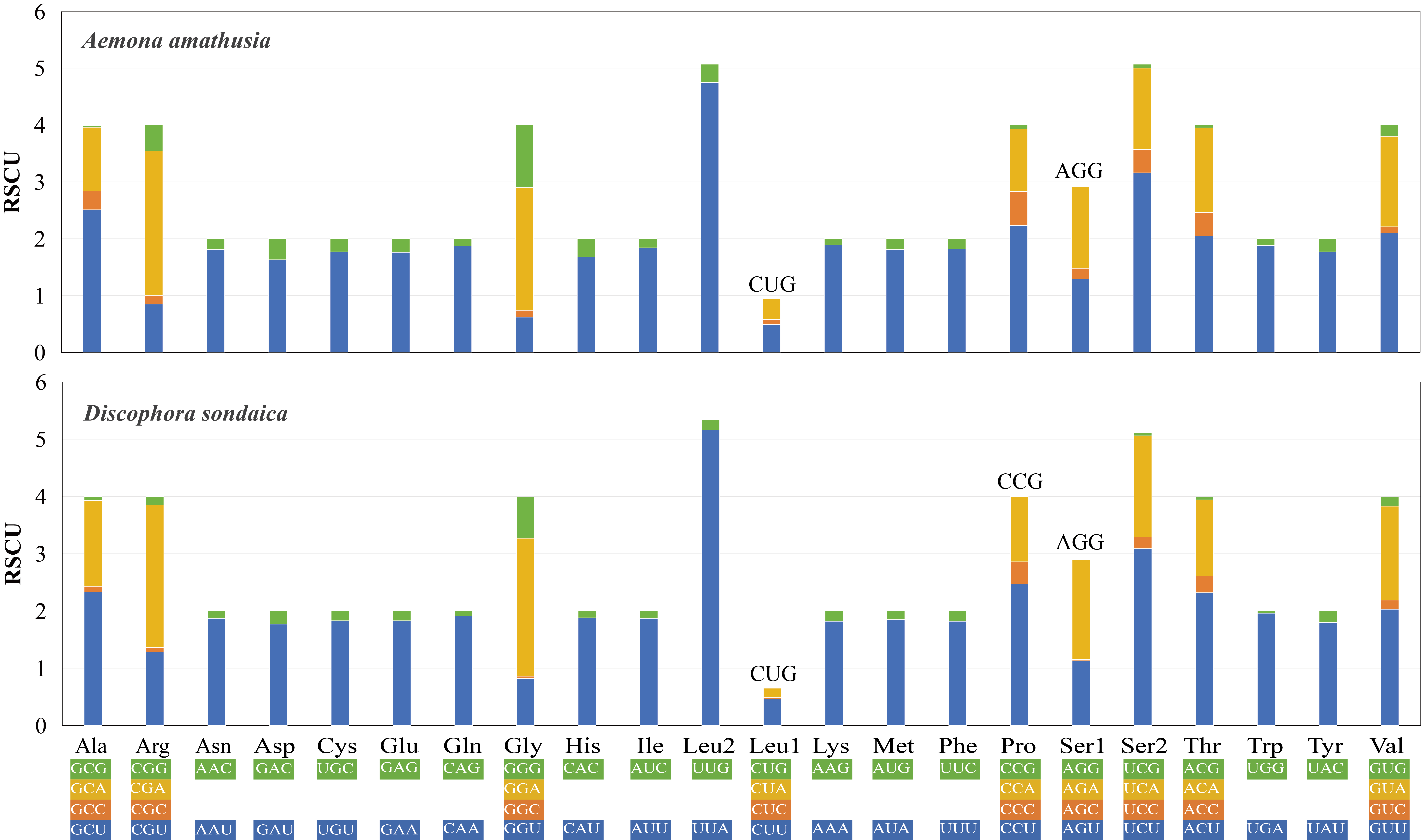

Supplement: Supplementary file 1 [file genes-16-00447-s001.zip › Figure S5.png]

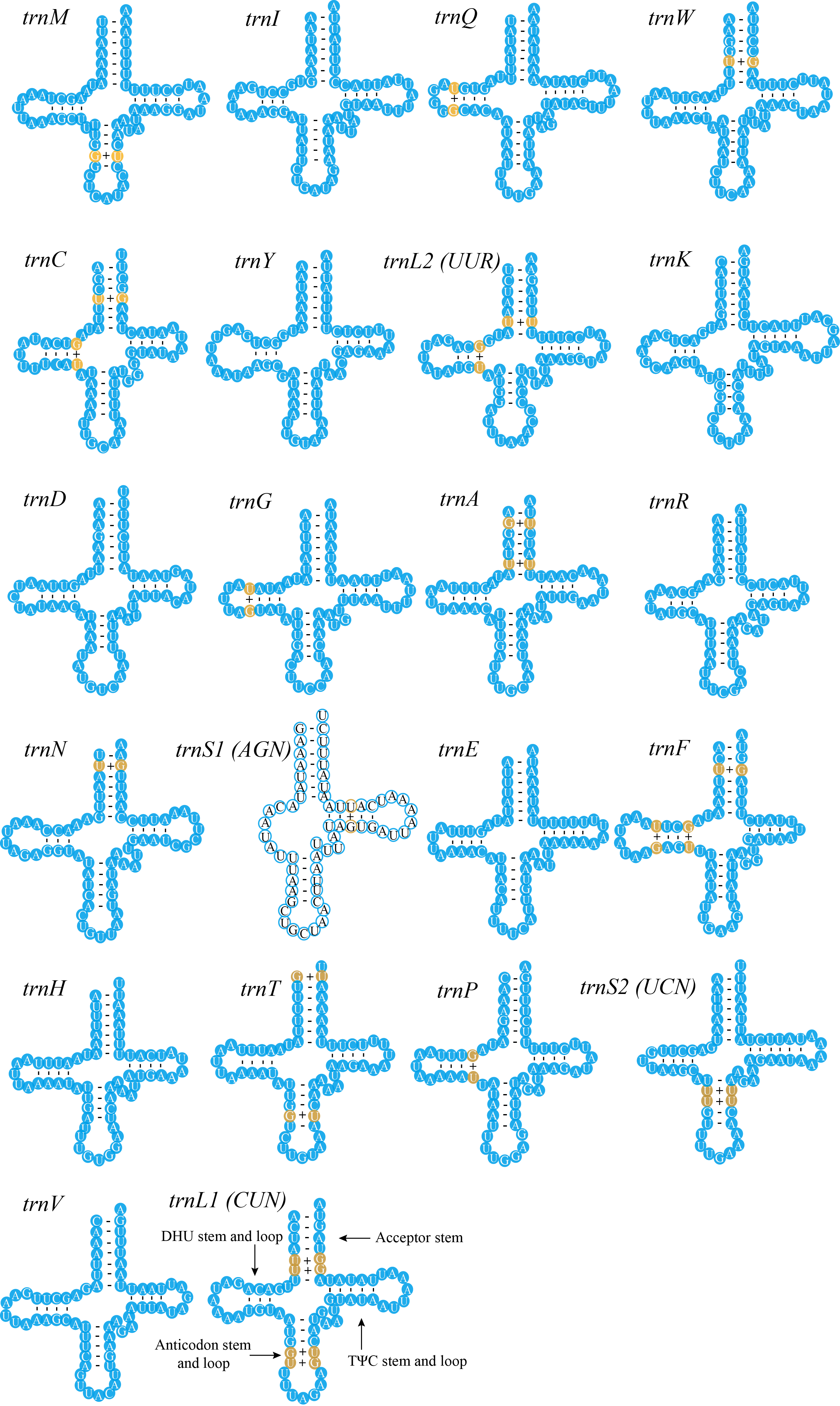

Supplement: Supplementary file 1 [file genes-16-00447-s001.zip › Figure S6.png]

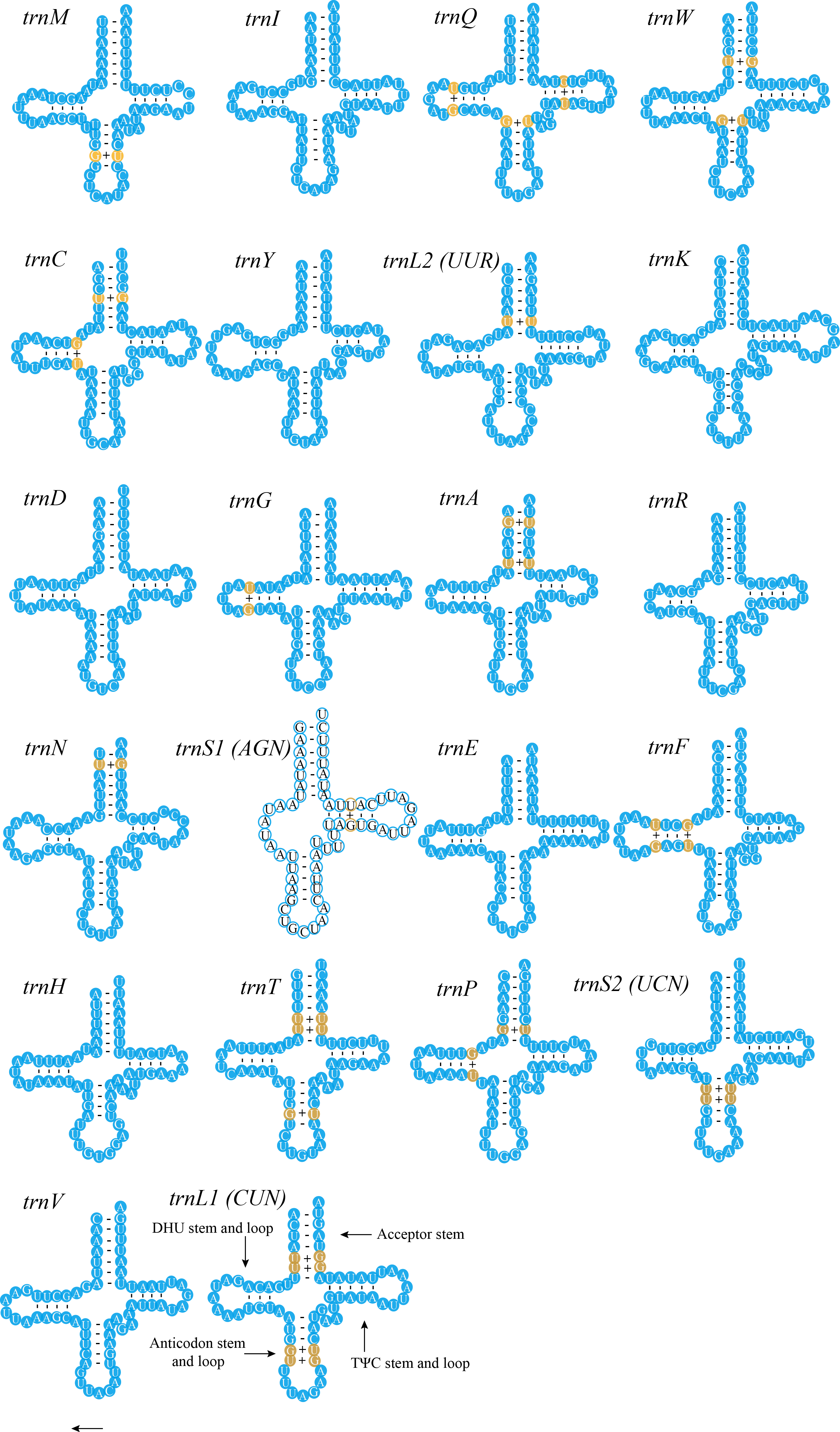

Supplement: Supplementary file 1 [file genes-16-00447-s001.zip › Figure S7.png]

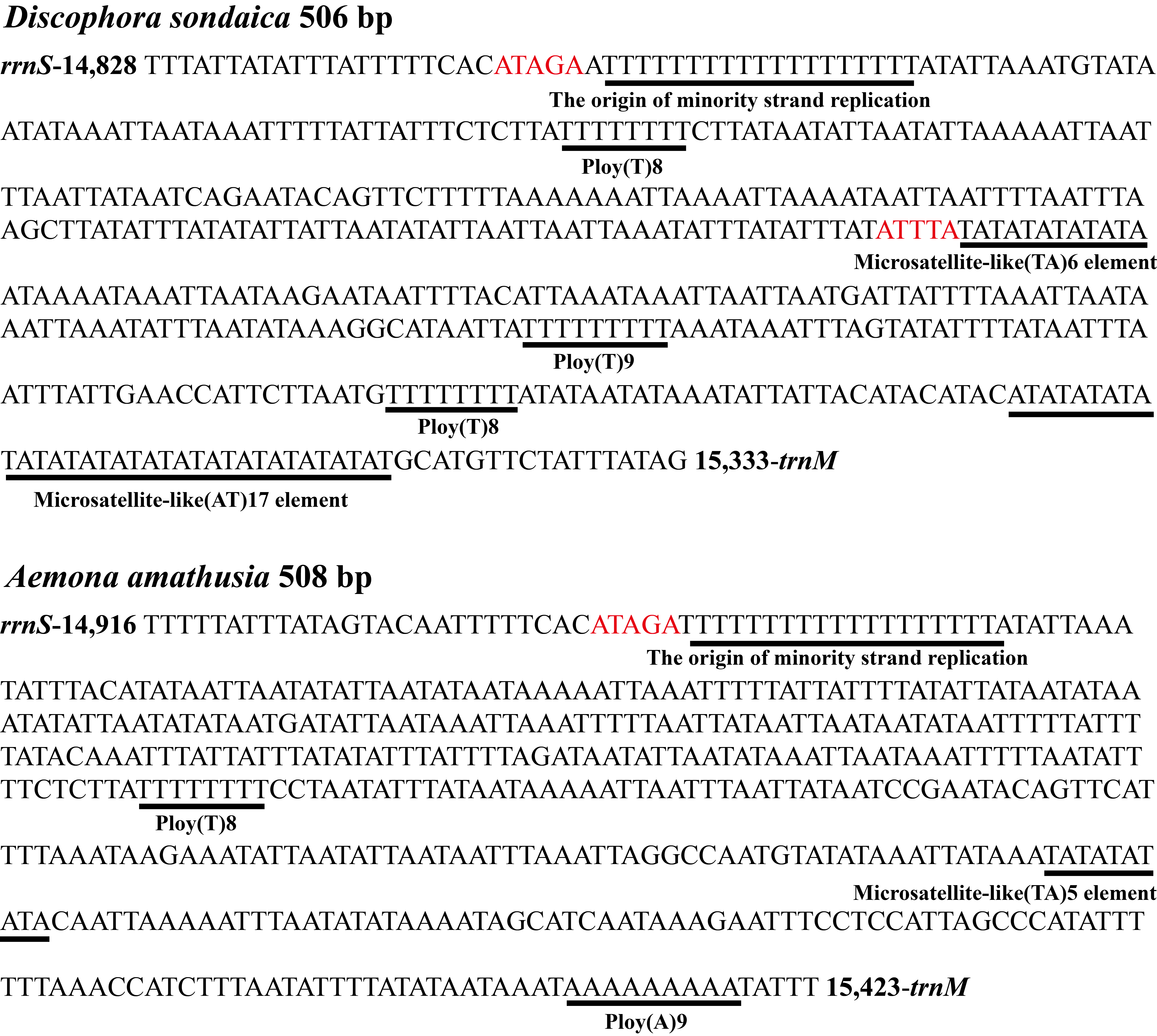

Supplement: Supplementary file 1 [file genes-16-00447-s001.zip › Figure S8.png]
